# Probing lithium mobility at a solid electrolyte surface

**DOI:** 10.1038/s41563-023-01535-y

**Published:** 2023-04-27

**Authors:** Clarisse Woodahl, Sasawat Jamnuch, Angelique Amado, Can B. Uzundal, Emma Berger, Paul Manset, Yisi Zhu, Yan Li, Dillon D. Fong, Justin G. Connell, Yasuyuki Hirata, Yuya Kubota, Shigeki Owada, Kensuke Tono, Makina Yabashi, Suzanne G. E. te Velthuis, Sanja Tepavcevic, Iwao Matsuda, Walter S. Drisdell, Craig P. Schwartz, John W. Freeland, Tod A. Pascal, Alfred Zong, Michael Zuerch

**Affiliations:** 1 University of Florida, Gainesville, FL USA; 2 Department of Chemistry, University of California, Berkeley, CA USA; 3 ATLAS Materials Science Laboratory, Department of Nano Engineering and Chemical Engineering, University of California, San Diego, La Jolla, CA USA; 4 Materials Sciences Division, Lawrence Berkeley National Laboratory, Berkeley, CA USA; 5 École Normale Supérieure – PSL, Paris, France; 6 Materials Science Division, Argonne National Laboratory, Lemont, IL USA; 7 National Defense Academy of Japan, Yokosuka, Japan; 8 RIKEN SPring-8 Center, Sayo, Hyogo Japan; 9 Japan Synchrotron Radiation Research Institute, Sayo, Hyogo Japan; 10 Institute for Solid State Physics, The University of Tokyo, Kashiwa, Japan; 11 Trans-scale Quantum Science Institute, The University of Tokyo, Tokyo, Japan; 12 Chemical Sciences Division, Lawrence Berkeley National Laboratory, Berkeley, CA USA; 13 Joint Center for Artificial Photosynthesis, Lawrence Berkeley National Laboratory, Berkeley, CA USA; 14 Nevada Extreme Conditions Laboratory, University of Nevada, Las Vegas, Las Vegas, NV USA; 15 X-ray Science Division, Argonne National Laboratory, Lemont, IL USA; 16 Materials Science and Engineering, University of California San Diego, La Jolla, CA USA; 17 Sustainable Power and Energy Center, University of California San Diego, La Jolla, CA USA; 18 Fritz Haber Institute of the Max Planck Society, Berlin, Germany; 19 Friedrich Schiller University Jena, Jena, Germany

**Keywords:** Batteries, Chemical physics, Characterization and analytical techniques, Density functional theory

## Abstract

Solid-state electrolytes overcome many challenges of present-day lithium ion batteries, such as safety hazards and dendrite formation^1,2^. However, detailed understanding of the involved lithium dynamics is missing due to a lack of in operando measurements with chemical and interfacial specificity. Here we investigate a prototypical solid-state electrolyte using linear and nonlinear extreme-ultraviolet spectroscopies. Leveraging the surface sensitivity of extreme-ultraviolet-second-harmonic-generation spectroscopy, we obtained a direct spectral signature of surface lithium ions, showing a distinct blueshift relative to bulk absorption spectra. First-principles simulations attributed the shift to transitions from the lithium 1 *s* state to hybridized Li-*s*/Ti-*d* orbitals at the surface. Our calculations further suggest a reduction in lithium interfacial mobility due to suppressed low-frequency rattling modes, which is the fundamental origin of the large interfacial resistance in this material. Our findings pave the way for new optimization strategies to develop these electrochemical devices via interfacial engineering of lithium ions.

## Abstract (web summary)

Understanding lithium dynamics in solid-state electrolytes used for Li-ion batteries can be challenging. Using nonlinear extreme-ultraviolet spectroscopies, a direct spectral signature of surface lithium ions showing a distinct blueshift relative to the bulk absorption spectra is observed in a prototypical solid-state electrolyte.

## Main

**Fig. 1:**
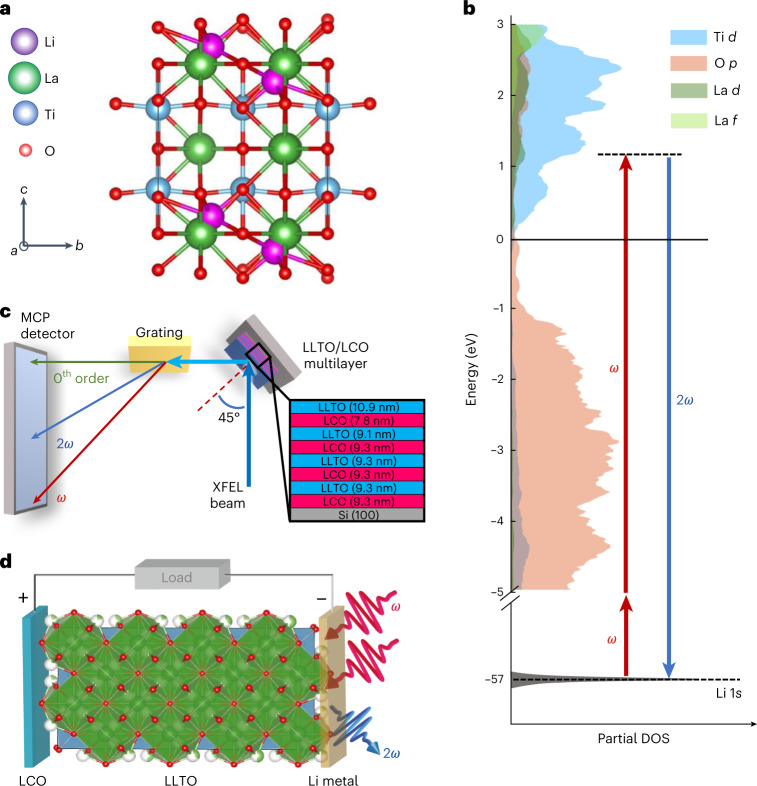
LLTO structure and experimental geometry. **a**, Basic crystal structure of LLTO, consisting of alternating Li-rich and -poor layers and Ti and O octahedra. **b**, Calculated partial density of states (DOS) for LLTO and indicated transitions for the XUV-SHG probe. **c**, Overview of the experimental setup used for measurement of XUV-SHG data in reflection geometry. The inset shows the layered sample structure with repeating layers of LLTO and LCO. **d**, Schematic representation of an LCO–LLTO stack forming a prototypical battery, with the XUV-SHG process indicated on the top surface; note that LLTO is polycrystalline in the film measured despite the schematic representation. XFEL, X-ray free-electron laser. MCP, microchannel plate.

In the pursuit of finding new solutions to overcome safety hazards of liquid electrolyte batteries, lithium lanthanum titanium oxide (Li_3*x*_La_2/3-*x*_TiO_3_, LLTO) has emerged as a promising candidate material. LLTO is classified as an *AB*O_3_ perovskite material (*A* is Li, La; B is Ti; Fig. 1a), consisting of an alternating arrangement of La-rich and -poor layers (or, equivalently, lithium vacancy-poor and -rich layers)^3^. It is known to have one of the highest ionic conductivities for Li-ion-containing oxides (1 × 10^−3^ S cm^–1^), comparable to liquid electrolytes, and can participate in rapid Li^+^ ion transport^4^. Challenges in regard to effective application of this class of solid-state electrolytes relate to limitations in physical contact, interfacial impedances, instability to contact with lithium metal and mechanical stabilization due to issues including material fractures and impurities^5,6^. In such oxide electrolyte films with thicknesses close to the range for lithium ion battery separators, these challenges may become even more pronounced, further emphasizing chemo-electromechanics phenomena at the interfaces^7^. A better knowledge of both surface and interface characteristics is thus necessary to aid in determining material compositions and designs that can effectively overcome these limitations, ultimately improving battery performance. However, to date there are no experimental techniques that can directly probe the interface of these complex materials with atomic specificity. Thus, mechanistic understanding of the behaviour of lithium ions at the interface can be inferred only from traditional electrochemical measurements employing idealized equivalent circuit models^8,9^.

Extreme-ultraviolet-second-harmonic generation (XUV-SHG)^10^ can be used to retrieve spectral signatures that specifically contain the contribution of ions at complex interfaces. Second-harmonic-generation occurs when two light waves of frequency *ω*, called the fundamental, are absorbed by the material to emit a wave at twice the frequency, 2*ω* (Fig. 1b). SHG intensity is proportional to the square of light intensity and the second-order susceptibility, *χ*^(2)^, where *χ*^(2)^ contains information related to the dielectric environment^11^. In addition, under the electric dipole approximation, the second-order susceptibility is only nonzero for systems lacking inversion symmetry, allowing SHG to probe the surface characteristics of a material exhibiting inversion symmetry in bulk, as is the case for LLTO. Measurements in the XUV and soft X-ray regime are particularly attractive because they enable elemental selectivity^12^ and have been achieved at free-electron lasers (FELs), where soft X-ray and XUV-SHG have been successfully employed to study surfaces^11,13^ and bulk anisotropies^14,15^. In addition, XUV-SHG with a tightly focused table-top source has recently been reported^16^. Of course the measured second-order susceptibility is generally small, rendering the generation and detection of SHG difficult—measurements require intense, coherent laser sources.

To understand how specific Li surface and interfacial structures can impact ion transport at LLTO surfaces, in this work we experimentally compare surface-specific properties of LLTO probed by XUV-SHG with bulk properties probed by X-ray absorption spectroscopy (XAS) derived from reflectivity measurements (see Methods). This work complements previous studies of Li using bulk-sensitive techniques such as cryogenic electron microscopy^17^. Similar to XUV-SHG, XAS probes transitions between occupied and unoccupied states, providing information about the local atomic and electronic structure of Li within the material but with the bulk signal dominating the response. By comparing the features at *ω* of the XAS spectrum with those at 2*ω* in the XUV-SHG spectrum—keeping in mind the different transitions that would occur, governed by the selection rules of these two processes—differences in bulk and surface characteristics can be determined. Spectral shifts at the lithium K-edge for both techniques were observed and interpreted using first-principles electronic structure simulations. Further ab initio molecular dynamics simulations revealed that lithium dynamics are substantially suppressed at the surface due to symmetry breaking, resulting in reduced lithium entropy and mobility that are visible in the XUV-SHG, and hence the large interfacial resistance in this material.

The sample considered here consists of alternating layers of LLTO and lithium-cobalt oxide (LCO) (Fig. 1c), representing a model solid-state battery (Fig. 1d). LCO is one of the most commercially successful cathode materials while LLTO is a widely used electrolyte^18^. Bulk absorption spectra in the XUV range were retrieved from linear XUV reflectivity measurements and contain contributions from both LLTO and LCO. The surface spectra on the same sample were measured using XUV-SHG. The thickness of the topmost LLTO layer (~11 nm) prevents access to the first buried interface of the polycrystalline LLTO–LCO stack using XUV-SHG. The discussion and analysis in this work are focused on the bulk and surface of the top-layer LLTO, relevant to describing the anode (lithium metal)/electrolyte interface (see Supplementary Sections 1–6 for additional discussion*)*. For selective addressing of lithium ions and measurement of the surface spectrum, the FEL is tuned across the lithium K*-*edge (61.3 eV) at half-resonance in the range 29–33 eV. The intensities of the fundamental FEL pulse, *I*(*ω*), and the second-harmonic signal *I*(2*ω*) are simultaneously recorded using an imaging spectrometer.

**Fig. 2:**
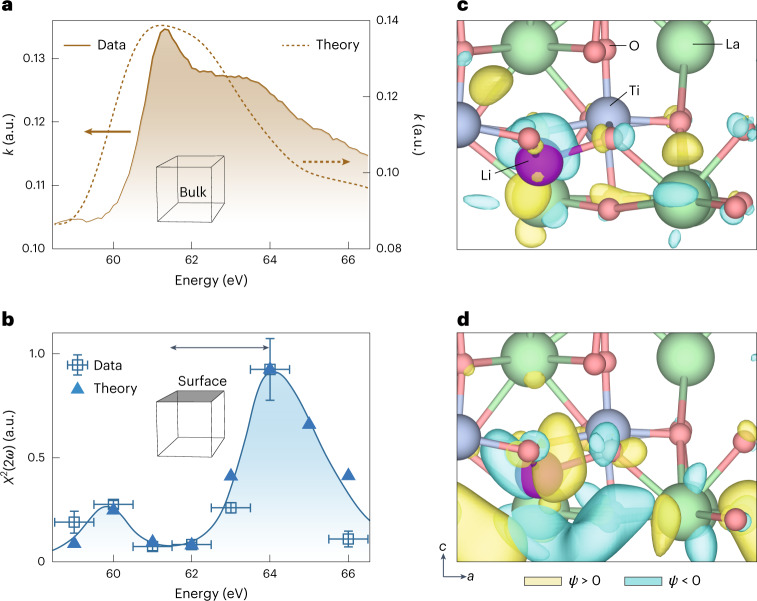
Measured and numerically simulated linear and nonlinear response of LLTO at the lithium K-edge. **a**, The measured imaginary part of the refractive index of LLTO (brown shaded area, left axis) agrees well with the numerically retrieved linear response (dashed brown line, right axis) around the Li K-edge that appears around 61 eV in LLTO. The calculated curve is an equal-weight linear superposition of response from both LLTO and LCO. See Supplementary Fig. 13 for the effect of including the LCO contribution. The slight disagreement of the linear response at higher energies could have stemmed from broadening due to unrealistic sample structures in the simulation, where an ideal crystal geometry is assumed. **b**, Experimentally derived second-order nonlinear susceptibility *χ*^(2)^(2*ω*) response across the Li K-edge (blue open squares, blue solid line for visual clarity). The computationally simulated second-order nonlinear susceptibility *χ*^(2)^(2*ω*) for a LLTO at the surface is denoted by solid triangles, and is in good agreement with the measurements. Vertical error bars correspond to errors in the quadratic fit of the second-order response; horizontal error bars are a result of energy jitter of the FEL for which 72,000 shots were collected at each photon energy. **a**,**b**, Double-sided arrow highlights the difference in peak positions between linear absorption (**a**) and second-harmonic response (**b**). **c**,**d**, Representative wave function of the resulting lithium atom core-excited states in the XUV-SHG spectrum at ~61 eV (**c**) and ~64 eV (**d**). We adopt the convention that the positive and negative phases of wave function are coloured beige and teal, respectively.

We find that the bulk linear absorption spectrum peaks at approximately 61.5 eV and monotonically decays with increasing energy (Fig. 2a). By contrast, the *χ*^(2)^(2*ω*) spectrum features a peak around 64 eV (Fig. 2b). Due to selection rules governing the SHG process, which can be viewed as the product of two dipole-allowed transitions—Li 1 *s* → *np* → (*n* + 1)*s*/*d*^19^—it is expected that lithium 1 *s* core electrons have allowed transitions only to unoccupied Li *s* final states, which may be hybridized with transition metal *d* states. It is also expected that the valence band is occupied and thus not available for transitions. We confirm these hypotheses by performing first-principles, velocity-gauge, real-time, time-dependent density functional theory (DFT) calculations^20^ with a numerical atomic orbital basis set to propagate the electronic structure of LLTO under an intense laser field (Supplementary Section 7). Our calculated spectrum is in excellent agreement with the experimental findings. Specifically, the first low-intensity feature on the XUV-SHG at 60 eV is due to transitions to Li 2 *s* surface states that are delocalized due to hybridization with unoccupied Ti 3 *d* states (Fig. 2c and Supplementary Figs. 7 and 8). Similarly, the main XUV-SHG peak at 64 eV is due to transitions to Li 3 *s* states but has higher intensity due to greater overlap between the core-excited state and the Lithium 1 *s* orbital (Fig. 2d). We underscore that the ~2 eV blueshift in the XUV-SHG spectrum is due to the inherent physics of the techniques, where XUV-SHG selection rules prohibit transitions to the unoccupied Li *p* states available to XAS rather than the dichotomy between surface and bulk properties. Indeed our simulations indicate that, were measurement possible, the XAS of the LLTO surface would be 0.2 eV red-shifted compared with the bulk spectrum (Supplementary Fig. 9).

**Fig. 3:**
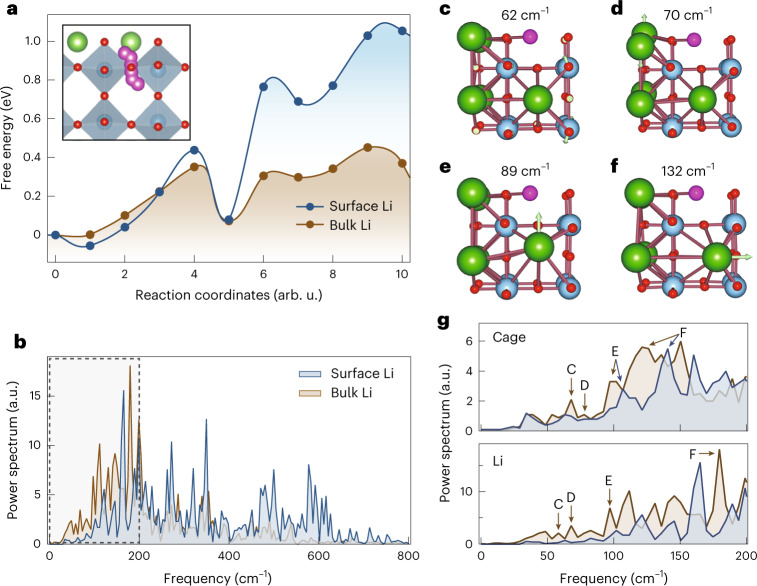
Restricted lithium dynamics at LLTO surfaces. **a**, Plot of the barrier for Li migration along the low-energy pathway (inset), resolved for Li atoms at the surface (blue curve) and in the bulk (brown curve). Lower barriers are calculated for bulk diffusion. **b**, Lithium vDoS energy distribution at the surface and in bulk LLTO. We found a reduction in the population of low-frequency rattling modes (50–100 cm^–1^) for the surface lithium ion, which resulted in 40% reduction in entropy. The region in the dashed rectangle is enlarged in the bottom panel of **g**. **c**–**f**, Visualization of bulk LLTO vibrational modes at 62 cm^–1^ (**c**), 70 cm^–1^ (**d**), 89 cm^–1^ (**e**) and 132 cm^–1^ (**f**). Arrows indicate the general direction of atom displacement at a particular frequency. Li vibrational dynamics are coupled with cage breathing modes (**c**,**f**), as well as the optical longitudinal (**d**) and transverse (**e**) modes. **g**, vDoS of the LLTO cage (top) and Li (bottom) vibrations in the low-frequency range. **c**–**f**, The various vibrational modes are indicated. Surface cages show suppressed or blueshifted vibrational modes, which compromises Li interfacial dynamics.

Previous reports have shown that XUV-SHG spectroscopy is very sensitive to local chemical environment and bonding chemistry^13^. Thus, the low variance in our experimental XUV-SHG spectrum and the excellent agreement with simulations (Fig. 2b), where we used a DFT relaxed static structure, indicates that lithium mobility at the surface is substantially restricted. We tested this hypothesis by means of further ab initio simulations. First, we found that the barrier for Li migration from one LTO cage to another is substantially higher at the surface than in the bulk (Fig. 3a). Nudged elastic band calculations reveal a barrier for Li diffusion, which follows a curved path that avoids empty *A* sites of ~0.2 eV in the bulk, in good agreement with previous reports^21,22^. This barrier increases to nearly 1 eV near the surface. Moreover, ab initio molecular dynamics simulations at 298 K reveal that Li mobility within the LTO cage is substantially more confined at the surface than in the bulk (Supplementary Figs. 10–13). In particular, the phonon vibrational density of states (vDoS) of surface lithium atoms shows a depopulation of modes of 50–150 cm^−1^ (0.67–0.22 ps) compared with the bulk (Fig. 3b). Further analysis of the LLTO vDOS reveals that, in the bulk, Li motions are strongly coupled with LLTO cage vibrations (Fig. 3c–g). For example, we find several normal modes that affect lithium mobility, primarily: (1) a mode at 62 cm^−1^ due to TiO_6_ octahedral rotation (Fig. 3c); (2) various optical modes (near 70 and 89 cm^−1^, respectively) due to La displacement (Fig. 3d,e); and (3) a combined La/TiO_6_ breathing mode near 132 cm^−1^ (Fig. 3f and Supplementary Videos 1–4). At the surface, due to inversion symmetry breaking, several of these lattice vibrations are either suppressed or shifted to higher frequencies, such as cage modes E and F shown in Fig. 3g. The suppression of vibrational modes at the material surface, while not a widely explored phenomenon, has been observed at various clays^23^. From the Debye theory of solids^24^, entropy is inversely proportional to the exponential of the frequency so that a blueshift of vDoS at the surface corresponds to lower entropy. Quantitatively, the suppression and blueshift of low-frequency modes at the surface of LLTO led to a calculated entropy of Li atoms of 18.1 J mol^–1^ K^–1^, ~40% lower than the bulk value.

Our findings provide a first crucial step towards long-sought-after probes for monitoring of lithium interfacial dynamics in operando. In particular, the calculated decrease in Li surface mobility and conductivity based on atomic simulations, which is consistent with spectroscopic measurements, motivates future studies aimed at probing more complex interfaces such as LLTO–LCO, and presently provides a rational basis for understanding one of the main obstacles regarding this class of solid-state electrolytes. LLTO is known to have high grain boundary resistance^25,26^, and difficulties in achieving high interfacial mobility impede its total ionic conductivity and effective use in batteries. It is commonly assumed that increased interfacial impedance results from the formation of a highly disordered interphase layer at the contact point between LLTO and the electrode^18^. With this in mind, various methods of reducing interfacial impedance have been investigated including surface coating^27^, buffer layer introductions^28^ and interface softening^29^. Our current results, suggesting reductions in lithium interfacial mobility at the surface due to intrinsic changes in LLTO cage vibrational modes, provide an additional design principle. We also note that anode-free solid-state batteries are an area of increasing interest^30^ and, although the interface between a solid electrolyte and a nonreactive current collector such as Cu is not technically 'free', from a chemical standpoint the mobility of Li in LLTO at the LLTO surface will not be meaningfully perturbed by such a configuration. Therefore, the mobility of Li at the free LLTO surface is highly relevant when considered for anode-free operation of solid-state batteries—in particular, for understanding how this initial mobility will direct subsequent Li metal plating/stripping reactions. We envision future optimization strategies based on manipulation of surface phonon modes, possibly through interface engineering and characterization of interfacial morphology by XUV-SHG. Such an approach will present a powerful new paradigm for enhancing the properties of complex material interfaces in general, which is complementary to other interface- and surface-sensitive spectroscopic approaches.

## Methods

### Experimental methods

The XUV-SHG measurements were conducted at BL1 of the SACLA FEL, under vacuum at ambient temperature. Data were collected using a 0.8 μm Al filter to prevent sample damage. The FEL *p*-polarized pulses with 30 fs pulse duration and energies ~13 µJ per pulse became incident with the LLTO–LCO multilayer at 45° relative to the surface normal, with a spot size of ~50 μm measured in full-width at half-maximum. Due to the FEL beam being incident at Brewster’s angle, it was heavily attenuated, with any reflectivity observed due to either polarization contamination or imperfections in beam alignment. The reflected light, consisting of the weak fundamental FEL beam and XUV-SHG signal, passed through a 200-µm horizontal slit before being directed onto a grating at 87° with respect to the surface normal. The beam was dispersed using a diffraction grating with 1,200 grooves mm^–1^ before collection on a microchannel plate detector (Rectangular, Hamamatsu Photonics) coated with CsI. Fundamental photon energies from the FEL were tuned at 28–33 eV in 0.5 eV steps. The resulting images were captured by camera (IPX-VGA120-LMCN, Imperx, Inc.). Shot-to-shot fluctuations of fundamental intensity were used to retrieve second-order susceptibility, and the slight photon energy jitter (approximately 0.2%) was used to increase spectral resolution.

The XAS spectra were obtained from XUV reflectivity measurements conducted at the Lawrence Berkeley National Laboratory Advanced Light Source at beamline 6.3.2. Details can be found in Supplementary Section 2.

### Data processing

The reflected intensity of each FEL shot was measured on the microchannel plate detector as a two-dimensional image containing the specular reflection of the grating, fundamental (*I*_*ω*_) and second-harmonic (*I*_2*ω*_) signals. At each photon energy, for a specific attenuation filter, approximately 60,000 shots were collected. After merging into a single variable, sections containing the primary features from the two-dimensional detector image were isolated. Pixels were vertically binned and Gaussian functions fit to the fundamental peak in each shot. For each peak position, poor-quality shots were identified as those having an *r*^2^ value for the fit <0.9. Outstanding shots were background corrected using the featureless portion of the spectrum that was averaged and subtracted. After initial processing, the data were merged and binned with respect to the fundamental intensity. Within each bin there was a sufficient number of shots used to extract the average intensity of the fundamental (*I*_*ω*_) peaks. The same procedure was carried out for the second-harmonic signal within each binned spectrum. The trapezoidal rule was used to integrate over the Gaussian fit of the second-harmonic signal to obtain the respective intensities. A quadratic function was fit to the data points of (*I*_*ω*_) versus (*I*_2*ω*_) at each incident pulse energy, to extract nonlinear susceptibility *χ*^(2)^(2*ω*) as a function of photon energy. Details can be found in Supplementary Section 6.

### Sample

The multilayer sample used in this study was composed of four repeating units of LLTO and LCO layers grown by pulsed-laser deposition on Si(100). Using X-ray photoelectron spectroscopy (XPS), it was determined that the top layer exhibited a composition of Li_0.09_La_0.64_TiO_3_. However, because XPS has poor sensitivity to Li and small errors in La:Ti ratios yield large errors in Li concentration estimates, a more accurate test of composition was performed. The films were digested with aqua regia solution for 3 days to ensure complete dissolution. The resulting supernatant was diluted with 3% HNO_3_ for analysis by inductively coupled plasma mass spectrometry (ICP–MS). All measurements used either Thermo iCAP Q ICP–MS or Thermo iCAP RQ ICP–MS. This resulted in a stoichiometry of Li_0.41_Li_0.53_TiO_3_ (see Supplementary Section 3 for XPS and ICP–MS measurements and additional discussion). The top layer is LLTO, which is ~11 nm in thickness. See Supplementary Fig. 4 for hard X-ray reflectivity measurements that enabled determination of the layer structure represented in the inset of Fig. 1c. Densities were determined by neutron reflection (Supplementary Fig. 5).

### Simulated XUV-SHG spectrum

The frequency-dependent linear response and nonlinear second-harmonic susceptibility of LLTO were assessed by real-time, velocity-gauge, time-dependent DFT^20^. A 2 × 2 × 2 super cell (38 total atoms) of the fundamental unit cell of the LLTO perovskite structure, Li_0.375_La_0.56_TiO_3_, with lattice constants *a* = 7.828 Å, *b* = 7.754 Å and *c* = 7.871 Å, was utilized. The electronic structure was described as a linear combination of localized atomic orbitals as implemented in the *Siesta* code, using a custom double-*ζ-*quality basis set. Real-space mesh energy cutoff was set to 5,226 eV, and a timestep of 0.04 a.u. (1.935 as) was used to propagate the system. The system was sampled at a 5 × 5 × 5 Г-centred *k*-point grid. A small impulse function was used to excite the system to extract linear response. The resulting frequency-dependent dielectric function was then obtained from Fourier transform of current density *J*(*t*). For further details on computational methods and techniques, the reader is referred to Supplementary Sections 7–13.

## Online content

Any methods, additional references, Nature Portfolio reporting summaries, source data, extended data, supplementary information, acknowledgements, peer review information; details of author contributions and competing interests; and statements of data and code availability are available at 10.1038/s41563-023-01535-y.

## Supplementary information

Supplementary Information Additional discussion and materials are provided in Supplementary Sections 1–13 and Supplementary Figs. 1–14. Animations of phonon modes discussed are visualized in Supplementary Videos 1–4. In each video, the vibrational frequency of the mode is indicated, and the arrows indicate the main direction of displacement as a visual guide.

Supplementary Video 1 Rotations of the TiO_6_ octahedra at 62 cm^–1^.

Supplementary Video 2 An in-plane optical TO mode at 70 cm^–1^.

Supplementary Video 3 An out-of-plane optical LO mode at 89 cm^–1^.

Supplementary Video 4 LTO breathing mode at 132 cm^–1^.

## Data Availability

All data shown in the main text have been made available through Dryrad: 10.6078/D1N41X. All other datasets generated during and/or analysed during this study are available from the corresponding author on reasonable request. The DFT calculations presented in the paper were carried out using publicly available electronic structure codes (Methods).

## Code availability

Thermodynamics were obtained by post trajectory analysis of a code that implements the 2PT method. This code can be accessed from our GitHub repository (https://github.com/atlas-nano/2PT). Structures and VASP/SIESTA input files used for calculations have been deposited at https://github.com/atlas-nano/LLTO_XRAY-SHG.
