# Supplementary material for: Probing lithium mobility at a solid electrolyte surface
Source: Nat Mater. 2023 Apr 27;22(7):848–52. doi: 10.1038/s41563-023-01535-y (PMC10313518; doi:10.1038/s41563-023-01535-y)
Supplement: Supplementary file 1 — Additional discussion and materials are provided in Supplementary Sections 1–13 and Supplementary Figs. 1–14. Animations of phonon modes discussed are visualized in Supplementary Videos 1–4. In each video, the vibrational frequency of the mode is indicated, and the arrows indicate the main direction of displacement as a visual guide. [file 41563_2023_1535_MOESM1_ESM.pdf]

nature materials

Letter

https://doi.org/10.1038/s41563-023-01535-y

Probing lithium mobility at a solid electrolyte surface

In the format provided by the authors and unedited

Table of Contents:

S1. Extraction of refractive index from reflectivity measurements……………………………………………2

S2. Experimental setup for reflectivity measurement………………………………………………………. 2

S3. Quantification of the Li content by X-ray Photoelectron Spectroscopy and Inductively Coupled Plasma Mass Spectrometry……………………………………………………………………………….. 2

S4. XUV reflectivity spectra and attenuation length in LLTO……………………………………………… 3

S5. Neutron reflectivity measurements……………………………………………………………………….3

S6. Nonlinear susceptibility extraction……………………………………………………………………….. 4

S7. First-Principles Simulation of Linear and Nonlinear Susceptibility of LLTO……………………………4

S8. Determination of the Lithium Diffusion Barrier………………………………………………………..5

S9. Ab-initio Molecular Dynamics Simulations…………………………………………………………… 5

S10. Lithium Power Spectrum and Entropy Quantification…………………………………………………5

S11. Phonon analysis……………………………………………………………………………………..5

S12. Lithium Dynamics and Thermodynamics at LLTO Interface Analysis………………………………… 5

S13. First-principles Calculation of Linear Susceptibility of LCO/LLTO composite………………………...6

Supplementary Figures 1–14……………………………………………………………………………….. 7

Supplementary Videos…………………………………………………………………………………… 13

References…………………………………………………………………………………………… 13

S1. Extraction of refractive index from reflectivity measurements

The refractive index of a material consists of real and imaginary parts that allow for linking our measurements to the electronic structure of the multilayer system. This is especially useful in obtaining element-specific information regarding spin-states, orbital character, and optical properties of materials.[1] The complex refractive index can be described as $\tilde{n} = n + ik$ with n corresponding to the real part and k to the imaginary part. For *s*-polarization, the total reflectance can be derived from the Fresnel equations to:

$$R = \left| \frac{\cos\theta_i - \sqrt{\tilde{n}^2 - \sin\theta_i^2}}{\cos\theta_i + \sqrt{\tilde{n}^2 - \sin\theta_i^2}} \right|^2 e^{-\sigma^2 q^2} \qquad (1)$$

where the incident angle measured w.r.t. the surface normal is defined as θ_i, and $e^{-\sigma^2 q^2}$ is the Debye-Waller factor describing the attenuation of the reflected beam due to scattering. To extract the refractive index, a numerical nonlinear least-squares algorithm was employed using Eq. (1), as described by Kaplan et al.[1] Since the material properties of the sample are described by n and k, which are components of the complex refractive index ($\tilde{n}$), they are not a function of the angle and the values are not solvable using a single measurement. Therefore, the total reflectivity (R) was measured at the ALS as a function of the incident angle (10°, 20°, 30°, 45°) and photon energy so that the system becomes overdetermined allowing for the retrieval of n and k at each photon energy. While theoretically two angles are sufficient to solve the set of equations, using four different angles was chosen to reduce the error.

S2. Experimental setup for reflectivity measurement

The absolute reflectance of LLTO was gathered at beamline 6.3.2 at the Advanced Light Source (ALS) at Lawrence Berkeley National Laboratory, with the setup summarized in **Fig. S1**. XUV *s*-polarized light first passes through a 4-jaw aperture and is then focused in both the horizontal and vertical directions, before becoming incident onto a series of 80 mm plane varied-line spacing (VLS) gratings. This specific type of grating was chosen based on our desired energy range of 25–73 eV. After exiting a slit, the then monochromatic beam continues to a three-mirror order suppressor. The beam was focused down to a spot size of 10 μm × 300 μm (FWHM) onto the sample contained under vacuum in the reflectometer. The reflected X-ray yield is measured using a large area photodiode connected to a low-noise amplifier and an analog-digital converter. To obtain a reflectivity spectrum, the monochromator is scanned in 0.1 eV steps and the reflected yield is recorded. A spectrum of the beam directly incident on the photodiode was collected and used for background removal.

S3. Quantification of the Li content by X-ray Photoelectron Spectroscopy and Inductively Coupled Plasma Mass Spectrometry.

XPS measurements were performed using a Specs PHOIBOS 150 hemispherical energy analyzer with a monochromated Al K_α X-ray source (**Fig. S3A**). Charge neutralization was carried out for insulating LLTO samples using a low-energy flood gun (5 eV electron energy and 50 μA emission current). Survey spectra were measured using a pass energy of 40 eV at a resolution of 0.2 eV/step and a total integration time of 0.1 s/point. Core-level spectra were measured using a pass energy of 20 eV at a resolution of 0.05 eV/step and a total integration time of 0.5 s/ point. Quantification was performed using Scofield cross-sections with Shirley-type background subtraction. The O 1s core level was further deconvoluted using 70−30 Gaussian−Lorentzian peak shapes to isolate the oxygen content from the LLTO film and exclude additional signal from surface contaminants. As Li concentrations cannot be accurately measured due to overlap with the Ti 3p core level, the Li concentration was estimated using the ratio of La:Ti concentration and the nominal material stoichiometry of $Li_{3x}La_{(2/3-x)}TiO_3$. This yielded an approximate LLTO stoichiometry of $Li_{0.09}La_{0.64}TiO_3$, which is lower than the initially targeted composition of $Li_{0.33}La_{0.55}TiO_3$. However, the Li content from the LLTO formula relies on a factor of 3 multiple relative to the La vacancy content, and small errors in the measured La stoichiometry relative to Ti will result in large errors in the estimated Li content. As a result, XPS is insufficiently accurate to appropriately quantify the Li content of these LLTO films. To

more accurately measure the Li content in the thin films, we performed additional experiments utilizing ICP-MS of single layer, amorphous LLTO thin films grown using the same PLD target and growth conditions as the LLTO layers in our LLTO-LCO superlattices. Several replicates were performed, yielding an average Li:La ratio of 0.766 (**Fig. S3B**). This corresponds to a nominal film stoichiometry of $Li_{0.41}La_{0.53}TiO_3$, consistent with the target stoichiometry of $Li_{0.33}La_{0.55}TiO_3$.

S4. XUV reflectivity spectra and attenuation length in LLTO

An important consideration given the sample structure is the possibility of contributions from the first buried interface to the observed XUV-SHG signal. To estimate the penetration depth at half resonance to the Li K edge, i.e. photon energies ranging from 28-33 eV as used in the measurements, we assume a density of LLTO of 5.13 g/cm³. This value is the average value of all relaxed LLTO structure currently available in the *materials project* repository[2] with densities ranging from 4.68 to 5.99 g/cm² for variations of this structure. Using neutron reflectivity measurements, we determined that the topmost LLTO layer had a density of 4.4 g/cm^3 (see **Section S5**), hence we use a density of 4.5 g/cm³ to estimate the penetration depth using the CXRO database[3] with the nominal stoichiometry measured using XPS (**Section S3**). We find that the penetration depth is in the range of 9 nm considering 45° angle of incidence on the sample around 30 eV. With the first layer exhibiting 11 nm thickness, the intensity impinging at the first interface at half resonance can be estimated to be in the order of 10% of the intensity incident at the surface. Due to the quadratic scaling law of XUV-SHG, additional attenuation of the outbound XUV-SHG wave propagating back through the topmost LLTO layer, and likely overall lower XUV-SHG efficiency due to less broken symmetry it is reasonable to neglect contributions of the first buried layer to the observed XUV-SHG signal. However, in the linear response X-ray reflectivity mild contributions from the first buried LCO layer can be observed due to the increase in the XUV penetration and escape depth (~25 nm) at around 60–70 eV (**Fig. S2B**).

The observed Li-K absorption edge (**Fig. S2A,B**), is comparable to several reported literature values for lithium-containing electrolyte materials ranging from 58 to 62 eV.[4,5] However, a larger shift (~6 eV) is observed in comparison to the Li metal *K*-edge at 54.7 eV reported by the CXRO database,[3] consistent with the formal oxidation state of lithium being Li^+, as expected for LLTO. Bader charge analysis shows that the Li ion carries a +0.903*e* charge. The reduced electron density around the Li atom results in higher excitation energies.[6] At lower photon energies, the Ti $M_{2,3}$ edge has a small contribution to the overall spectrum from the peak located at 39 eV describing the transition from the $3p_{3/2,1/2}$ core states to the unoccupied higher level energy states.[5] The Co $M_{2,3}$ edge also appears to have a modest contribution to the spectrum with a peak located at 59 eV, close to the 58.9 eV reported by the CXRO database.[3] The broad spectral feature at approximately 48.3 eV is likely a result from the O L_1 edge typically located near 41.6 eV.[7] It has been found that decreased metal-oxygen bond covalency is directly related to decreased effective nuclear charge, with observed O L_1 edges for other metal oxides from 42 eV for NiO to 44 eV for Fe_2O_3.[8] The reported Bader charges for metal and oxygen pair in NiO are +1.30*e*/-1.30*e* while that of Fe-O pair charges in Fe_2O_3 are +1.84 e/-1.21e [Ref. 9,10]. Our calculation for the Ti-O pair in LLTO shows that the Bader charges are +2.10*e*/-1.20*e* but due to asymmetry in Li rich and poor region. The large deviation observed in LLTO would be consistent with the decreased covalency by a smaller effective nuclear charge from Ti in LLTO, compared to that of Fe in Fe_2O_3, continuing in the trend of increasing O L_1 edges observed. The O_1 edge of La has a small contribution to the spectrum with a peak located at 36 eV, close to its 34.3 eV recorded value.

We did not observe any sample damage either from within a single shot or upon repeated exposure. We believe these samples at these relatively low flux do not undergo changes due to radiation.

S5. Neutron reflectivity measurements

Neutron reflectivity experiments on a nominally identical LLTO/LCO multilayer were performed at room temperature on the Liquids Reflectometer (BL-4B) of the Spallation Neutron Source at Oak Ridge National Laboratory. The reflectivity as a function of momentum transfer Q (= $4\pi \sin\theta / \lambda$), as shown in **Fig. S5A**, was measured by choosing a select number of incident angles θ and using time of flight analysis of a range of neutron wavelengths λ. The wavelength band, with a fixed width of 3.4 Å, was adjusted between 2.55 Å and 16.7 Å. The measured reflected neutron intensities were normalized on direct beam intensity

measurements to obtain the reflectivity. Fitting the experimental reflectivity provided a depth profile of the neutron scattering length density, as shown in **Fig. S5B**. From the neutron scattering length density the density of the top LLTO is determined to be 4.4 g/cm^3.

S6. Nonlinear susceptibility extraction

From each XFEL shot, the 0th order, 1st order and 2nd order diffraction are all recorded. A Gaussian peak was then fit to each shot. Looking at the FWHM of the first diffraction, if it was outside of 1.5 standard deviations of the mean the shot was discarded. The location of the Gaussian peak was used to place each shot into bins by energy. The integral of the fundamental shot was used to sort shots into bins by incident intensity. The grating equation was used to find the absolute values of energy.

To extract the nonlinear susceptibility scaling with fundamental intensity for each energy, the shots in each bin were sub-binned based on incident fundamental intensity and then averaged. Then the average second harmonic intensity was found from each bin, and the corresponding intensities were calculating by integrating the Gaussians using the trapezoidal rule. Then the nonlinear susceptibility was extracted by fitting the second-order response.

The inherent photon energy and pulse intensity jitter of the SASE-FEL creates a dataset where FEL shots with similar photon energy can be grouped together following the procedure outlined in Ref. 11. The pulse intensity jitter at each grouped energy is then used to extract second-order susceptibility $\chi^{(2)}(2\omega)$ by fitting a second order function to $I(2\omega)$ over $I(\omega)$. This extraction protocol permits us to relate the nonlinear response of the material to the dielectric environment at the surface of the topmost LLTO layer.

S7. First-Principles Simulation of Linear and Nonlinear Susceptibility of LLTO

The frequency-dependent linear response and nonlinear second harmonic susceptibility of LLTO was assessed by means of real-time velocity-gauge time dependent density functional theory simulations.[12–15] A 2×2×2 super cell (38 total atoms) of the fundamental unit cell of LLTO perovskites structure, $Li_{-0.375}La_{0.625}TiO_3$,[16] with lattice constant a = 7.828 Å, b = 7.754 Å, c = 7.871 Å, was utilized. The electronic structure was described as a linear combination of localized atomic orbitals as implemented in the *Siesta* code.[17] A semi-core norm-conserving pseudopotential, that include the semi-core level electrons relevant to XUV excitation, was employed using as basis set of double-ζ quality:

Atom	Basis set
Li	{1s(2ζ), 2s(2ζ), 2p(2ζ), 3s(2ζ), 3d(2ζ)}
La	{5s(2ζ), 6s(2ζ), 5p(2ζ), 5d(2ζ)}
Ti	{3s(2ζ), 3p(2ζ), 4s(2ζ), 3d(2ζ), 4p(2ζ)}
O	{2s(2ζ), 2p(2ζ)}

The real space mesh energy cutoff was set to 5226 eV and the timestep of 0.04 a.u. (1.935 as) was used to propagate the system. The system was sampled at 5×5×5 Γ-centered k-point grid. For the linear adsorption calculations, a small impulse function was used to excite the system. The resulting frequency-dependent dielectric function was then obtained from a Fourier transform of the current density $J(t)$ (**Fig. S6A**). For the second order susceptibility, a 2.5 fs sine-squared envelope monochromatic pulse with varying frequency ranging from 28-32 eV, and intensity ranging from 10^{11}–10^{15} W/cm^2, were used to propagate the system. First, we propagated the bulk LLTO system under 30 eV pulse at 10^{14} W/cm^2 and observed no second harmonic response (**Fig. S6B**, red line). This is consistent with the expectation that a second harmonic response will only occur at the surface of LLTO, where the inversion symmetry is broken. Next, we simulate the surface response of LLTO by constructing a slab geometry, i.e., adding a 10-Å vacuum in the z-direction. In addition, we artificially broke the symmetry, to ensure that the SHG emerges only at the surface, by excluding the semi-core electrons from all layers except the top layer. Further details on this approach can be found in the work by Lam et al.[18]

Fig. S6B depicts the second harmonic response under 30 eV monochromatic perturbation (**Fig. S6B**, blue lines), which demonstrates that the second harmonic generation results from the surface LLTO layer only. To extract the second order susceptibility, the Fourier transformed current density $J(\omega)$ at 2ω was fitted to a quadratic equation of field strength, based on a Taylor expansion of the generalized nonlinear susceptibility equation:

$$\frac{P_i}{\varepsilon_0} = \sum_j \chi_{ij}^{(1)} E_j + \sum_{jk} \chi_{ijk}^{(2)} E_j E_k + \sum_{jkl} \chi_{ijkl}^{(3)} E_j E_k E_l + \cdots \quad (2)$$

The simulated SHG spectrum in the main text **Fig. 2B** is derived by varying the monochromatic perturbation and perform this fitting procedure. Additional simulations were performed to verify that there was no SHG signal from Li atoms in the center of our LLTO slab, while another simulation revealed that the intensity of the SHG signal varied almost linearly with the Li atom surface concentration.

S8. Determination of the Lithium Diffusion Barrier

We performed Nudged Elastic Band (NEB) calculations[19,20] along the Li migration path and compared the results between Li migration within the bulk and toward surface. All calculations were performed using the Vienna Ab-initio Simulation Package (VASP) using the NEB algorithm implemented with the software.[21] A total of 11 images were used to simulate the lithium migration path.

S9. Ab-initio Molecular Dynamics Simulations

Molecular dynamics simulation of bulk and slab LLTO was performed using VASP. The system was propagated with *k*-point sampling grid of 2×2×2 (2×2×1 for the slab system) with the temperature of the system controlled at 300 K using a Nose-Hoover thermostat. The equations of motion of the ion were numerically propagated forward in time using the Verlet algorithm, within at integration timestep of 0.5 fs. The system was allowed to equilibrate for 1 ps, followed by an additional 100 ps simulation for post-trajectory analysis. Snapshots 0.5 ps apart were also used to calculate the Bader charge of each different atoms in the system.[22] An additional system was simulated to provide further details of Li dynamics at the LLTO/Li metal interface. Here, 6 layers of Li metal (24 atoms) are placed in contact with the original LLTO slab, and the system was propagated in time in the same way as the LLTO bulk and LLTO slab systems.

S10. Lithium Power Spectrum and Entropy Quantification

Using the trajectories from our AIMD simulations, we quantified the Lithium entropy using the Two Phase-Thermodynamics (2PT) code. The details of the 2PT method have been presented elsewhere[23–25]; here we outline the salient points. We first calculated the density of states (DOS, also known as the spectral density) function as a Fourier transform of the atomic velocity autocorrelation function.[23,26] We then calculated the entropy by separately considering the diffusive and vibrational motions of the atoms. Notably, since the DOS is obtained directly from MD, the resulting entropy inherently contains contributions due to harmonic vibrational, as well as anharmonic liberational and self-diffusional motions.

S11. Phonon analysis

To get a better understanding of the LTO lattice vibrations, we performed phonon frequencies calculation using perturbation theory with VASP.[27,28] Here, the Hessian matrix was first determined, and the dynamical matrices were solved for dependent frequencies and eigenvectors. We considered lithium atom in the bulk (3D geometry) and at the surface (slab 2D geometry). We visualized each of the modes using VESTA[29] and the VASP-plot-modes[30] procedure.

S12. Lithium Dynamics and Thermodynamics at LLTO Interface Analysis

To provide further understanding to Li atoms dynamics at LLTO interfaces, we analyzed the trajectories from our AIMD simulations. We calculated the in-plane and out-of-plane Li atoms displacement in bulk and at the interface, where "out-of-plane" refers to the crystallographic *c*-axis. We found that the motions of Li in bulk are identical between in-plane and out-of-plane directions. On the other hand, out-of-plane motion of Li at the interface is greatly hindered (see **Fig. S11**). To quantify this effect, we calculated the entropy of Li and found that the entropy is lower at the interface, compared to the bulk. This corresponds to a "hardening" of vibrational modes, which manifested as a blueshift of the vibrational DOS. Lastly, we verified that the Li at interface of LLTO/Vacuum model was similar to LLTO/Li (the expected interface in a real battery) by calculating the entropy profile along the c-axis (see **Fig. S12**). We find that the entropy of the two models is identical, hence justifying our choice of the model in the linear and non-linear response calculations.

S13. First-principles Calculation of Linear Susceptibility of LCO/LLTO composite

Linear response of LCO was simulated at the same level of theory employed in Section S7. The lattice constant of LCO unit cell was chosen as 4.99721 Å. We then performed a linear combination between the resulting linear response spectra of LCO and LLTO (See **Fig. S13**). The resulting spectra was found to be in better agreement to the experiment, as expected since the experiment probes both material at the same time and therefore provide signal that is collective response of the two materials.

Supplementary Figures

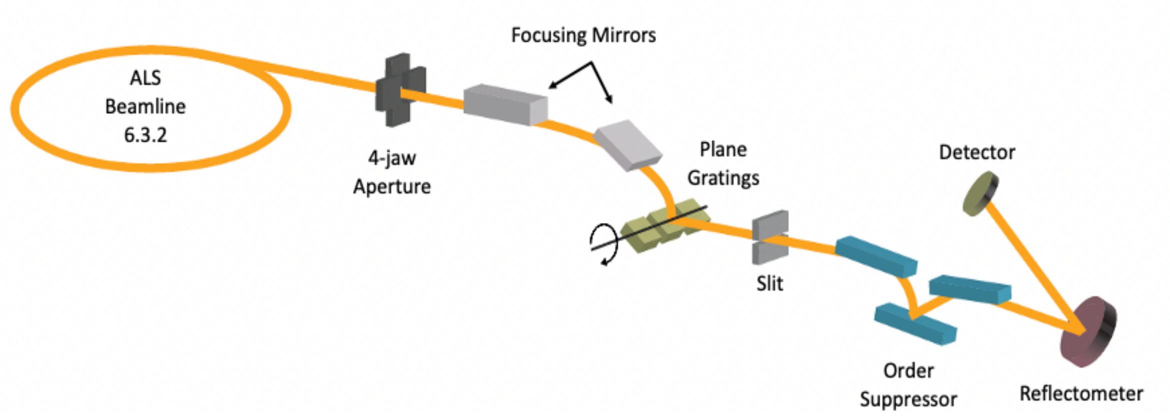

Figure S1. Beamline 6.3.2 at the Advanced Light Source (ALS). Illustrated above is the experimental geometry of the reflectivity experiments conducted at ALS. The incoming beam passes through a 4-jaw aperture prior to being steered by dual focusing mirrors onto a rotatable plane varied-line spacing (VLS) grating. Continuing, the reflected beam passes through a slit before entering an order suppressor setup. The XUV beam enters the reflectometer chamber and hits the sample at varying angles of incidence where the subsequent signal is collected on a detector perpendicular to the reflectometer surface. The grating-slit arrangement acts as monochromator for the broadband undulator radiation emerging from the ALS.

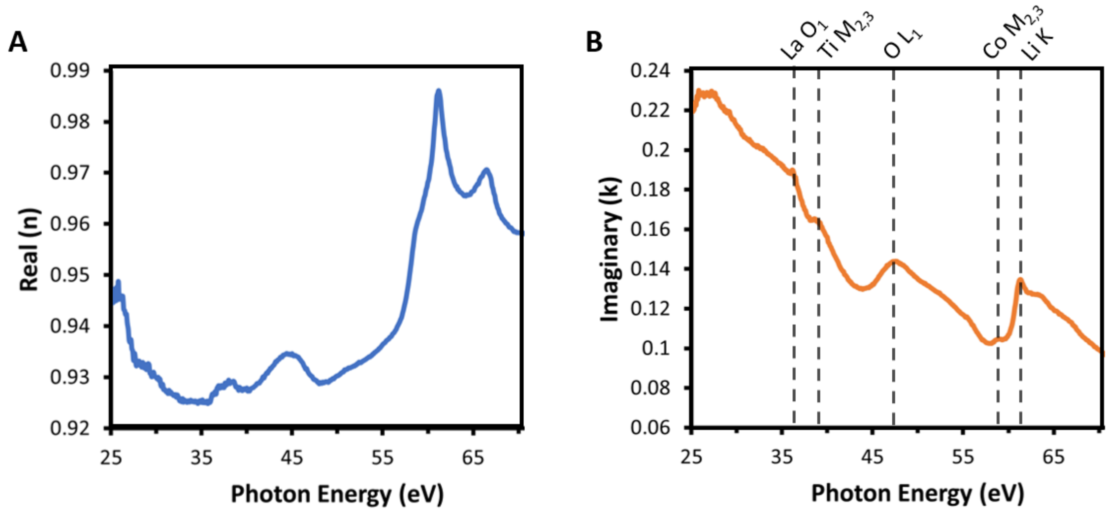

Figure S2. Retrieved complex refractive index of LLTO. Data was obtained from reflectivity measurements conducted at the ALS beamline 6.3.2. **A**, Retrieved real part of the refractive index *n*. **B**, Retrieved imaginary part of the refractive index *k* (orange). The dashed lines on each plot show the resonant photon energy of the Ti $M_{2,3}$ edge (39.0 eV), O L_1 edge (48.3 eV) Co $M_{2,3}$ edge (58.9 eV), and the Li *K*-edge (61.3 eV), respectively.

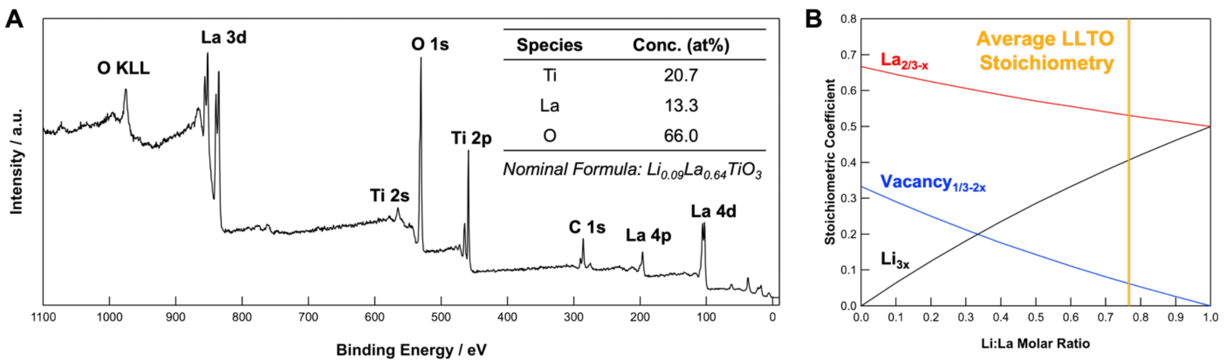

Figure S3. Quantification of Li Content in LLTO Films. A, The surface concentration of Li in the LLTO films were estimated by XPS. Because lithium overlaps with titanium at low binding energy, the amount of lithium must be calculated using the relative fraction of lanthanum vs. titanium. The total stoichiometry was determined to be $Li_{0.09}La_{0.64}TiO_3$ at the surface. **B**, ICP-MS measurements overlaid with variation in La, Li and vacancy content according to the formula $Li_{3x}La_{(2/3-x)}TiO_3$. ICP-MS measurements indicate the true thin film stoichiometry is closer to $Li_{0.41}La_{0.53}TiO_3$.

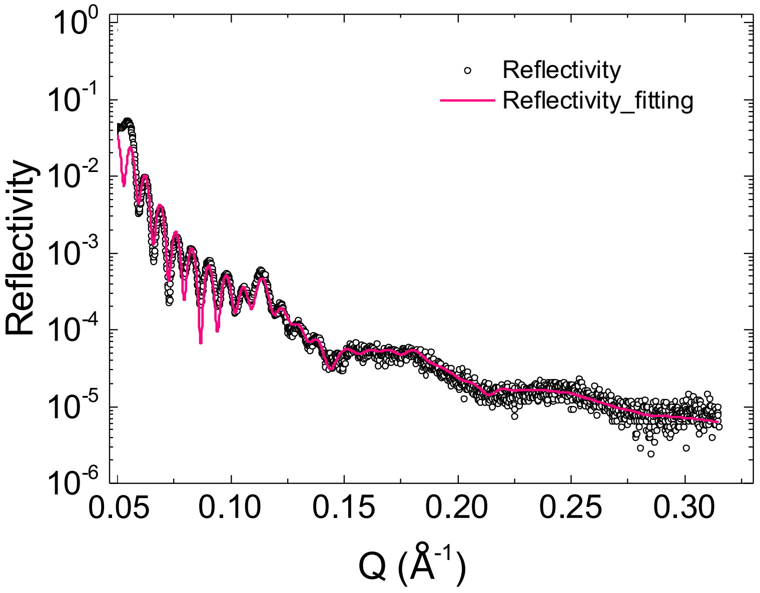

Figure S4. Hard X-ray reflectivity of the sample. The low-angle reflectivity shows oscillations from both the total film thickness (narrow fringes) and superlattice structure (wide fringes). The reflectivity was fit to determine the layer thicknesses shown in Figure 1 of the main text. X-ray reflectivity was measured on a Bruker D8 diffractometer with Cu K_α radiation. After performing geometrical corrections[31], the results were fit with the GenX program[32].

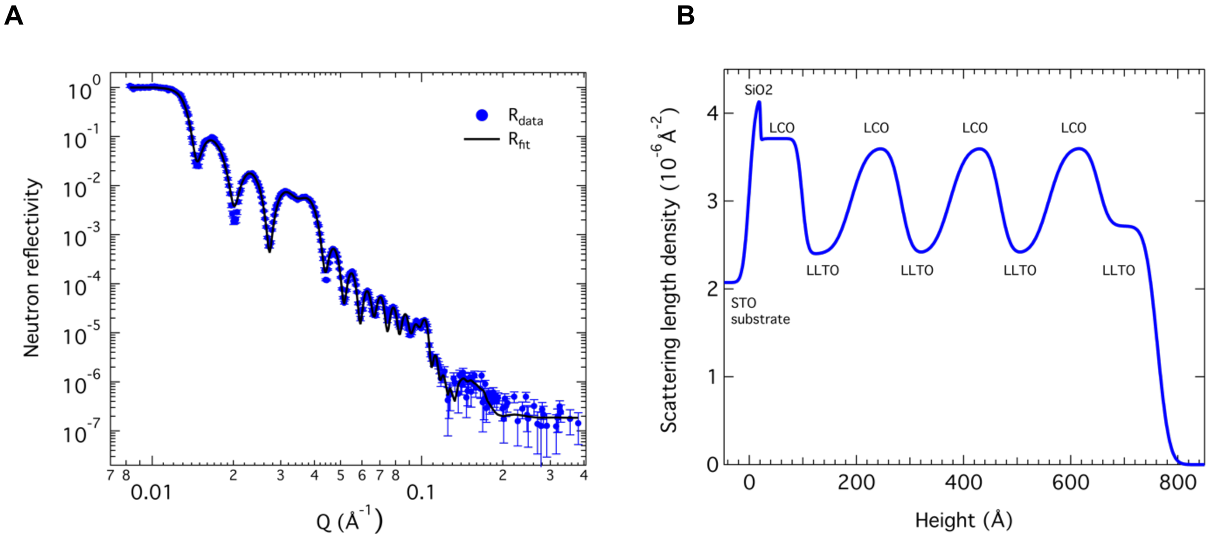

Fig S5. Neutron Reflectivity of the Sample. A, Neutron reflectivity data (circular markers) measured on the Liquids Reflectometer as a function of momentum transfer Q and fit (line) with the GenX program.[32] Error bars indicate the statistical error. **B,** The neutron scattering length density as a function of height above the surface of the substrate determined from fitting the experimental neutron reflectivity. Only the real part of the scattering length density is shown as the imaginary part is three orders of magnitude weaker than the real part and does not play a significant role in determining the reflectivity. During fitting, the parameters (thickness, roughness, scattering length density) describing of the three central LLTO/LCO bilayers were fixed to be identical to each other, while the parameters for the bottom LCO and top LLTO were allowed to be different from the other LCO and LLTO layers, respectively.

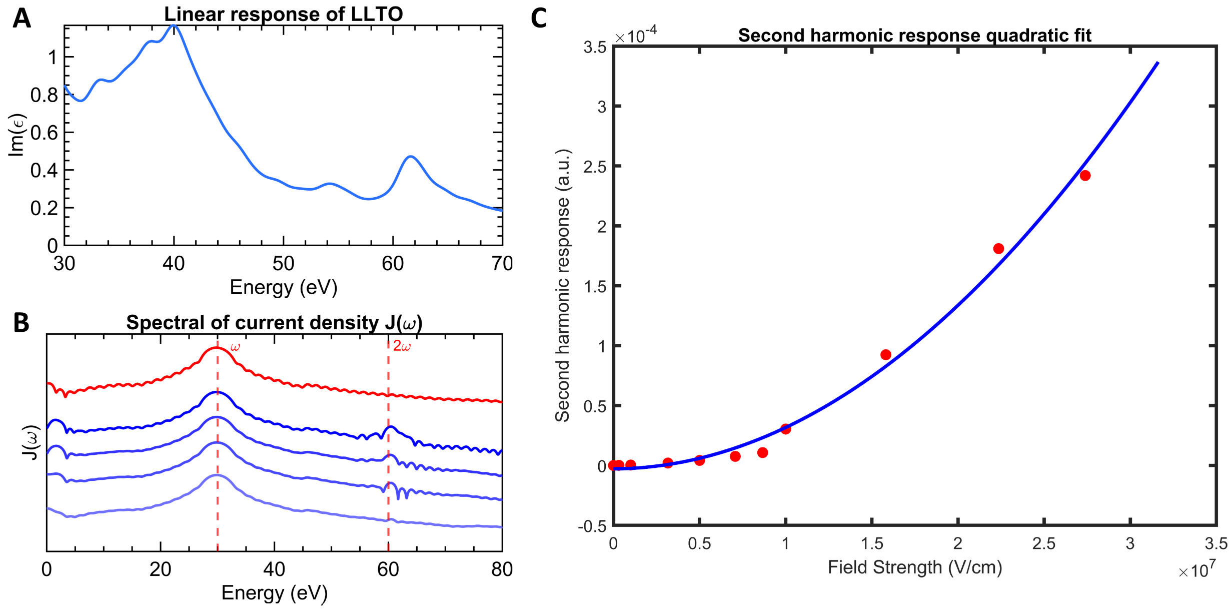

Figure S6. First-principles simulation of linear and nonlinear response of LLTO in the extreme ultraviolet. A, Calculated linear response spectrum of LLTO in the XUV. **B**, Spectrum of the current density $J(\omega)$ at a monochromatic perturbation at 30 eV at varying intensities showing the increasing second order (2ω) response for the inversion symmetry broken surface Li atoms (blue lines) at field strengths of 1×10^{11}, 1×10^{12}, 1×10^{13} and 1×10^{14} V/cm (bottom to top) that is not observed in bulk LLTO (red line – at a field strength of 1×10^{14} V/cm). **C,** Quadratic fit to field dependent for extraction of second order susceptibility.

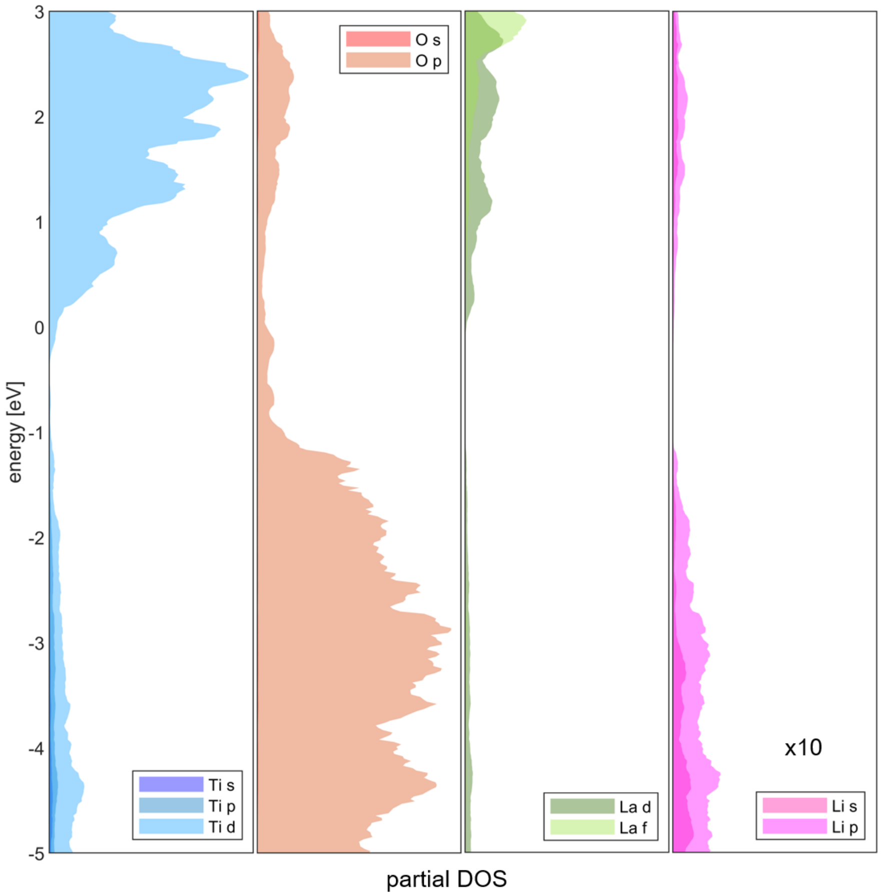

Figure S7. **Calculated partial density of states of LLTO for each element.** Ti *d*, O *p*, and La *d* and *f* states are identified as primary components forming the hybridized valence states shown in main text Fig. 1c.

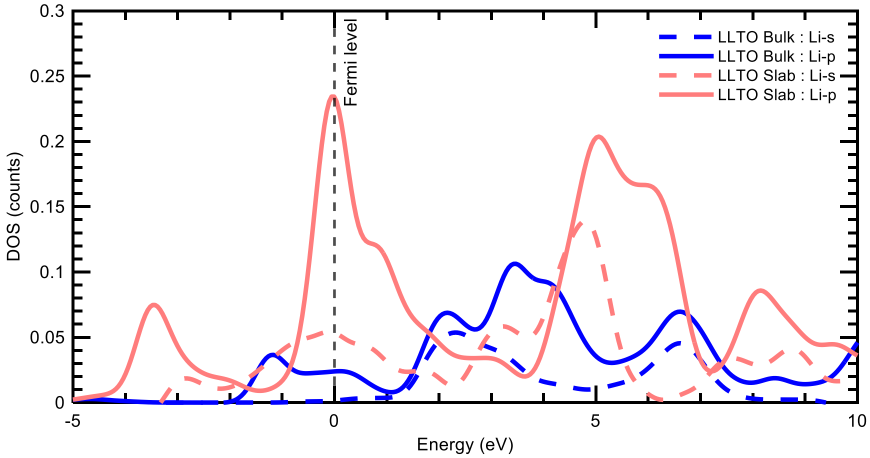

Figure S8. Projected density of state for LLTO, showing the energy distribution of the Lithium s (dashed lines) and p (solid lines) states. As before we separately consider and resolve the response from lithium atoms in the bulk (blue lines) and slab (red lines) geometries.

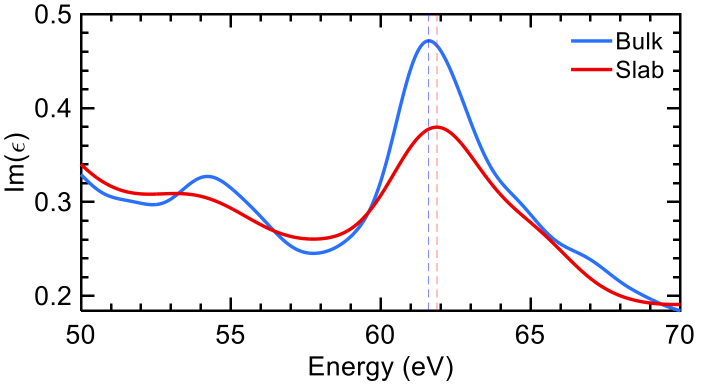

Figure S9. Calculated x-ray linear adsorption spectra of LLTO. We separately consider and resolve the response from lithium atoms in the bulk (3D) and slab (2D) geometries. We find that the slab spectrum is red-shifted by 0.2 eV compared to the bulk.

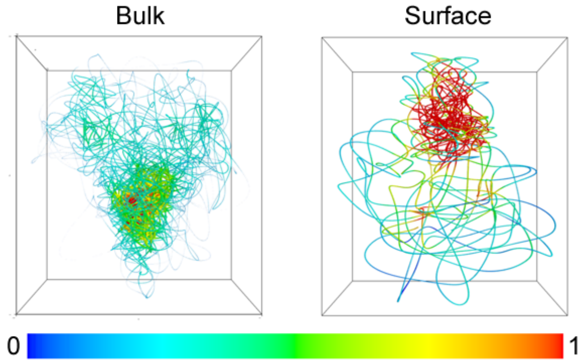

Figure S10. Distribution of lithium displacement in the LTO cage from AIMD simulations. Results for lithium atoms in the bulk (3D geometry) and surface (2D slab geometry) are presented. The color bar indicates the relative probabilities.

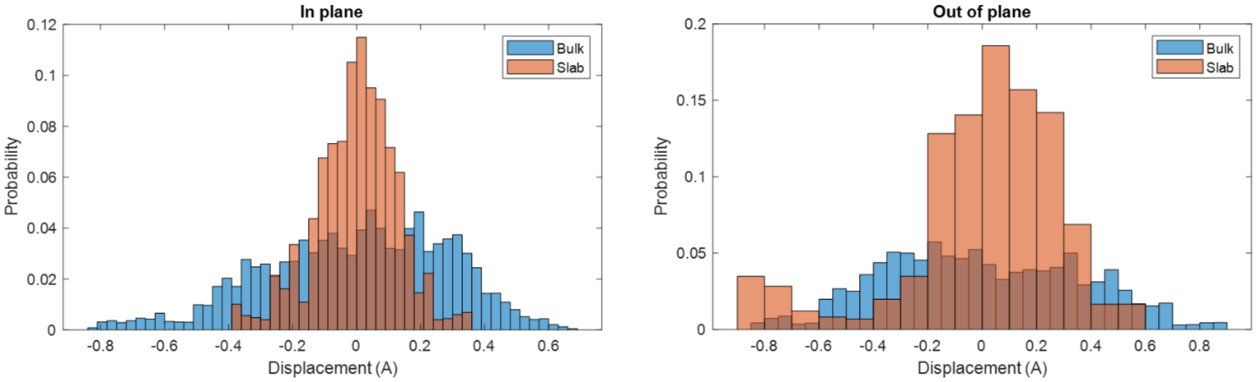

Figure S11. **Population analysis of lithium displacement from the center of the LTO cage from AIMD simulations**. The dynamics of Lithium atoms in the bulk (blue) and at the surface (orange) is presented for displacement in the XY plane (left panel) and out-of-plane (right panel).

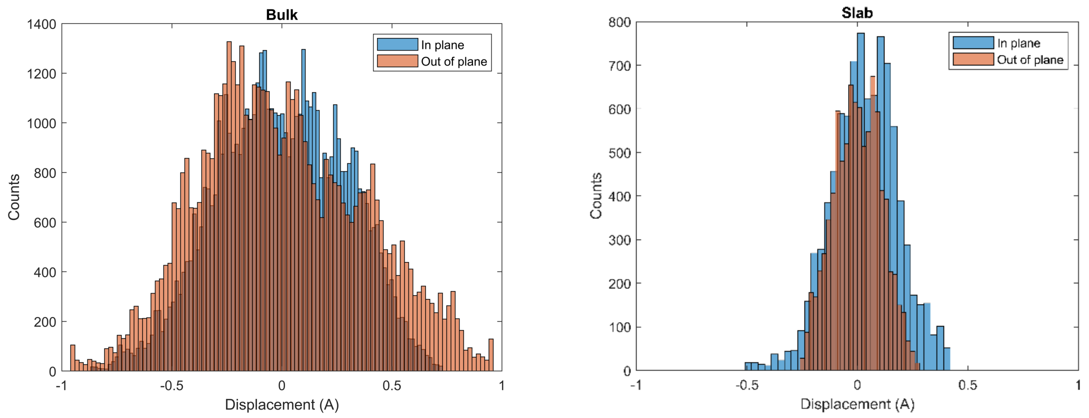

Figure S12. Displacement of bulk and surface lithium in LLTO from AIMD simulations. The Li mobility in the bulk (left) and surface (right) are compared for both in-plane (blue) and out-of-plane (brown) motions, where "out-of-plane" refers to the direction parallel to the crystallographic *c*-axis. In the bulk, Li motion is very similar between in-plane and out-of-plane motion. On the other hand, the Li at the interface sees reduction in its ability to travel. Additionally, the out-of-plane motion (motion perpendicular to the surface) is greatly hindered relative to the bulk.

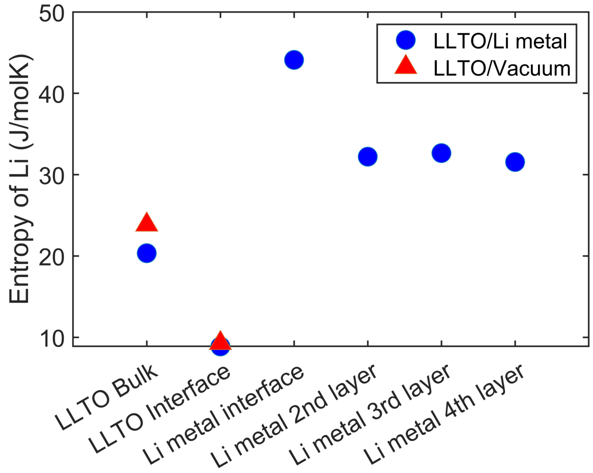

Figure S13. Comparison of the Li dynamics at the LLTO/Vacuum and LLTO/Li metal interface. The dynamical behavior of Li at the LLTO interface is similar between the LLTO/Vacuum and LLTO/Li metal systems by computing the entropy profile of the Li atoms along the slab. As shown, the entropy of Li, and thus the dynamical behavior, near both interfaces is nearly identical, justifying our SHG measurements at the LLTO/Vacuum interface as being relevant for understanding the properties of the Li(anode)/LLTO interface in a real solid-state battery. Of note, we find that the entropy of Li atoms in the Li-metal quickly converge to the experimental value of ~29 J/mol/K.

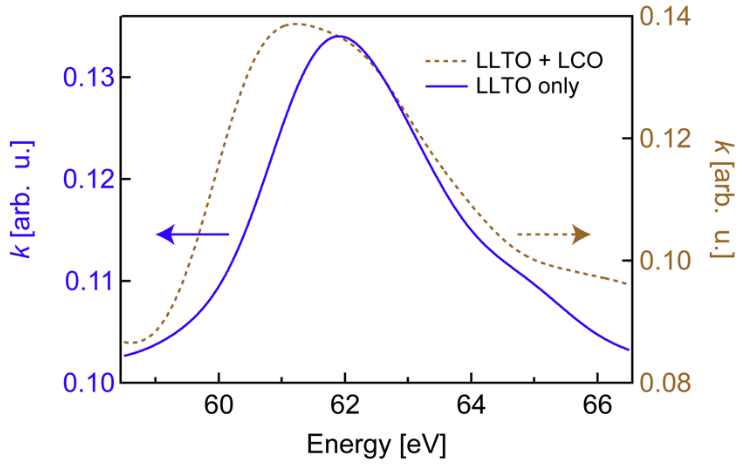

Figure S14. Effect of considering LCO in the linear absorption spectrum. In Fig. 2A in the main text, the theoretical linear absorption curve (reproduced here as dashed curve) considers the contribution from both LLTO and the underlying LCO layer, giving an excellent match in the peak position as the experimentally measured absorption peak. Here, we show the calculated absorption for the LLTO layer only (blue curve), which has a slight blue shift of the peak. This comparison indicates that it is necessary to consider both LLTO and LCO contributions in reproducing the experimental value in Fig. 2A.

Supplementary Videos **(supplied as separate files). Animated illustrations of low energy LLTO vibrational modes.**

The vibrational frequency of each mode is indicated, and the arrows indicate the main direction of displacement, to guide the eyes.

Supplementary Video 1: Rotations of the TiO_6 octahedra at 62 cm^{-1}.

Supplementary Video 2: An in-plane optical TO mode at 70 cm^{-1}.

Supplementary Video 3: An out-of-plane optical LO mode at 89 cm^{-1}.

Supplementary Video 4: LTO breathing mode at 132 cm^{-1}.

References

(1) Kaplan, C. J.; Kraus, P. M.; Gullikson, E. M.; Borja, L. J.; Cushing, S. K.; Zürch, M.; Chang, H.-T.; Neumark, D. M.; Leone, S. R. Retrieval of the Complex-Valued Refractive Index of Germanium near the M $_{4,5}$ Absorption Edge. *J. Opt. Soc. Am. B* **2019**, *36* (6), 1716. https://doi.org/10.1364/JOSAB.36.001716.

(2) Jain, A.; Ong, S. P.; Hautier, G.; Chen, W.; Richards, W. D.; Dacek, S.; Cholia, S.; Gunter, D.; Skinner, D.; Ceder, G.; Persson, K. A. Commentary: The Materials Project: A Materials Genome Approach to Accelerating Materials Innovation. *APL Mater.* **2013**, *1* (1), 011002. https://doi.org/10.1063/1.4812323.

(3) Henke, B. L.; Gullikson, E. M.; Davis, J. C. X-Ray Interactions: Photoabsorption, Scattering, Transmission, and Reflection at E = 50-30, 000 EV, Z = 1-92. *At. Data Nucl. Data Tables* **1993**, *54* (2), 181–342. https://doi.org/10.1006/adnd.1993.1013.

(4) Ma, C.; Chen, K.; Liang, C.; Nan, C.-W.; Ishikawa, R.; More, K.; Chi, M. Atomic-Scale Origin of the Large Grain-Boundary Resistance in Perovskite Li-Ion-Conducting Solid Electrolytes. *Energy Environ. Sci.* **2014**, *7* (5), 1638. https://doi.org/10.1039/c4ee00382a.

(5) Saitoh, M.; Gao, X.; Ogawa, T.; Ikuhara, Y. H.; Kobayashi, S.; Fisher, C. A. J.; Kuwabara, A.; Ikuhara, Y. Systematic Analysis of Electron Energy-Loss near-Edge Structures in Li-Ion Battery Materials. *Phys. Chem. Chem. Phys.* **2018**, *20* (38), 25052–25061. https://doi.org/10.1039/C8CP03390K.

(6) Pascal, T. A.; Boesenberg, U.; Kostecki, R.; Richardson, T. J.; Weng, T.-C.; Sokaras, D.; Nordlund, D.; McDermott, E.; Moewes, A.; Cabana, J.; Prendergast, D. Finite Temperature Effects on the X-Ray Absorption Spectra of Lithium Compounds: First-Principles Interpretation of X-Ray Raman Measurements. *J. Chem. Phys.* **2014**, *140* (3), 034107. https://doi.org/10.1063/1.4856835.

(7) Thompson, A. C.; Attwood, D. T.; Howells, M. R.; Kortright, J. B.; Robinson, A. L.; Underwood, J. H.; Kim, K.-J.; Kirz, J.; Lindau, I.; Pianetta, P.; Winick, H.; Williams, G. P.; Scofield, J. H. X-Ray Data Booklet. CXRO, Berkeley (**2009**).

(8) Biswas, S.; Husek, J.; Londo, S.; Baker, L. R. Highly Localized Charge Transfer Excitons in Metal Oxide Semiconductors. *Nano Lett.* **2018**, *18* (2), 1228–1233. https://doi.org/10.1021/acs.nanolett.7b04818.

(9) Neufeld, O.; Toroker, M. C. Platinum-Doped α-Fe_2O_3 for Enhanced Water Splitting Efficiency: A DFT+U Study. *J. Phys. Chem. C* **2015**, *119* (11), 5836–5847. https://doi.org/10.1021/jp512002f.

(10) Ralph, C.; Lemoine, P.; Boyd, A.; Archer, E.; McIlhagger, A. The Effect of Fibre Sizing on the Modification of Basalt Fibre Surface in Preparation for Bonding to Polypropylene. *Appl. Surf. Sci.* **2019**, *475*, 435–445. https://doi.org/10.1016/j.apsusc.2019.01.001.

(11) Uzundal, C. B.; Jamnuch, S.; Berger, E.; Woodahl, C.; Manset, P.; Hirata, Y.; Sumi, T.; Amado, A.; Akai, H.; Kubota, Y.; Owada, S.; Tono, K.; Yabashi, M.; Freeland, J. W.; Schwartz, C. P.; Drisdell, W. S.; Matsuda, I.; Pascal, T. A.; Zong, A.; Zuerch, M. Polarization-Resolved Extreme-Ultraviolet Second-Harmonic Generation from $LiNbO_3$. *Phys. Rev. Lett.* **2021**, *127* (23), 237402. https://doi.org/10.1103/PhysRevLett.127.237402.

(12) Hohenberg, P.; Kohn, W. Inhomogeneous Electron Gas. *Phys. Rev.* **1964**, *136* (3B), B864–B871. https://doi.org/10.1103/PhysRev.136.B864.

(13) Yabana, K.; Bertsch, G. F. Time-Dependent Local-Density Approximation in Real Time. *Phys. Rev. B* **1996**, *54* (7), 4484–4487. https://doi.org/10.1103/PhysRevB.54.4484.

(14) Bertsch, G. F.; Iwata, J.-I.; Rubio, A.; Yabana, K. Real-Space, Real-Time Method for the Dielectric Function. *Phys. Rev. B* **2000**, *62* (12), 7998–8002. https://doi.org/10.1103/PhysRevB.62.7998.

(15) Pemmaraju, C. D.; Vila, F. D.; Kas, J. J.; Sato, S. A.; Rehr, J. J.; Yabana, K.; Prendergast, D. Velocity-Gauge Real-Time TDDFT within a Numerical Atomic Orbital Basis Set. *Comput. Phys. Commun.* **2018**, *226*, 30–38. https://doi.org/10.1016/j.cpc.2018.01.013.

(16) Qian, D.; Xu, B.; Cho, H.-M.; Hatsukade, T.; Carroll, K. J.; Meng, Y. S. Lithium Lanthanum Titanium Oxides: A Fast Ionic Conductive Coating for Lithium-Ion Battery Cathodes. *Chem. Mater.* **2012**, *24* (14), 2744–2751. https://doi.org/10.1021/cm300929r.

(17) Soler, J. M.; Artacho, E.; Gale, J. D.; García, A.; Junquera, J.; Ordejón, P.; Sánchez-Portal, D. The SIESTA Method for Ab Initio Order-N Materials Simulation. *J. Phys. Condens. Matter* **2002**, *14* (11), 2745. https://doi.org/10.1088/0953-8984/14/11/302.

(18) Lam, R. K.; Raj, S. L.; Pascal, T. A.; Pemmaraju, C. D.; Foglia, L.; Simoncig, A.; Fabris, N.; Miotti, P.; Hull, C. J.; Rizzuto, A. M.; Smith, J. W.; Mincigrucci, R.; Masciovecchio, C.; Gessini, A.; Allaria, E.; De Ninno, G.; Diviacco, B.; Roussel, E.; Spampinati, S.; Penco, G.; Di Mitri, S.; Trovò, M.; Danailov, M.; Christensen, S. T.; Sokaras, D.; Weng, T.-C.; Coreno, M.; Poletto, L.; Drisdell, W. S.; Prendergast, D.; Giannessi, L.; Principi, E.; Nordlund, D.; Saykally, R. J.; Schwartz, C. P. Soft X-Ray Second Harmonic Generation as an Interfacial Probe. *Phys. Rev. Lett.* **2018**, *120* (2), 023901. https://doi.org/10.1103/PhysRevLett.120.023901.

(19) Henkelman, G.; Jóhannesson, G.; Jónsson, H. Methods for Finding Saddle Points and Minimum Energy Paths. In *Theoretical Methods in Condensed Phase Chemistry*; Schwartz, S. D., Ed.; Progress in Theoretical Chemistry and Physics; Springer Netherlands: Dordrecht, 2002; pp 269–302. https://doi.org/10.1007/0-306-46949-9_10.

(20) Henkelman, G.; Uberuaga, B. P.; Jónsson, H. A Climbing Image Nudged Elastic Band Method for Finding Saddle Points and Minimum Energy Paths. *J. Chem. Phys.* **2000**, *113* (22), 9901–9904. https://doi.org/10.1063/1.1329672.

(21) Kresse, G.; Furthmüller, J. Efficient Iterative Schemes for Ab Initio Total-Energy Calculations Using a Plane-Wave Basis Set. *Phys. Rev. B* **1996**, *54* (16), 11169–11186. https://doi.org/10.1103/PhysRevB.54.11169.

(22) Tang, W.; Sanville, E.; Henkelman, G. A Grid-Based Bader Analysis Algorithm without Lattice Bias. *J. Phys. Condens. Matter* **2009**, *21* (8), 084204. https://doi.org/10.1088/0953-8984/21/8/084204.

(23) Lin, S.-T.; Blanco, M.; Goddard, W. A. The Two-Phase Model for Calculating Thermodynamic Properties of Liquids from Molecular Dynamics: Validation for the Phase Diagram of Lennard-Jones Fluids. *J. Chem. Phys.* **2003**, *119* (22), 11792–11805. https://doi.org/10.1063/1.1624057.

(24) Lin, S.-T.; Maiti, P. K.; Goddard, W. A. Two-Phase Thermodynamic Model for Efficient and Accurate Absolute Entropy of Water from Molecular Dynamics Simulations. *J. Phys. Chem. B* **2010**, *114* (24), 8191–8198. https://doi.org/10.1021/jp103120q.

(25) Pascal, T. A.; Lin, S.-T.; Iii, W. A. G. Thermodynamics of Liquids: Standard Molar Entropies and Heat Capacities of Common Solvents from 2PT Molecular Dynamics. *Phys. Chem. Chem. Phys.* **2010**, *13* (1), 169–181. https://doi.org/10.1039/C0CP01549K.

(26) Berens, P. H.; Mackay, D. H. J.; White, G. M.; Wilson, K. R. Thermodynamics and Quantum Corrections from Molecular Dynamics for Liquid Water. *J. Chem. Phys.* **1983**, *79* (5), 2375–2389. https://doi.org/10.1063/1.446044.

(27) Ackland, G. J.; Warren, M. C.; Clark, S. J. Practical Methods Inab Initiolattice Dynamics. *J. Phys. Condens. Matter* **1997**, *9* (37), 7861–7872. https://doi.org/10.1088/0953-8984/9/37/017.

(28) Baroni, S.; de Gironcoli, S.; Dal Corso, A.; Giannozzi, P. Phonons and Related Crystal Properties from Density-Functional Perturbation Theory. *Rev. Mod. Phys.* **2001**, *73* (2), 515–562. https://doi.org/10.1103/RevModPhys.73.515.

(29) Momma, K.; Izumi, F. VESTA 3 for Three-Dimensional Visualization of Crystal, Volumetric and Morphology Data. *J. Appl. Crystallogr.* **2011**, *44* (6), 1272–1276. https://doi.org/10.1107/S0021889811038970.

(30) Rehn, D. Generate VESTA Mode Files from VASP OUTCAR Files, 2021. https://github.com/rehnd/VASP-plot-modes (accessed 2021-12-19).

(31) Gibaud, A.; Vignaud, G.; Sinha, S. K. The Correction of Geometrical Factors in the Analysis of X-Ray Reflectivity. *Acta Crystallogr. A* **1993**, *49* (4), 642–648. https://doi.org/10.1107/S0108767392013126.

(32) Björck, M.; Andersson, G. GenX: An Extensible X-Ray Reflectivity Refinement Program Utilizing Differential Evolution. *J. Appl. Crystallogr.* **2007**, *40* (6), 1174–1178. https://doi.org/10.1107/S0021889807045086.
