# Supplementary figures and images for: Probing lithium mobility at a solid electrolyte surface
Source: Nat Mater. 2023 Apr 27;22(7):848–52. doi: 10.1038/s41563-023-01535-y (PMC10313518; doi:10.1038/s41563-023-01535-y)

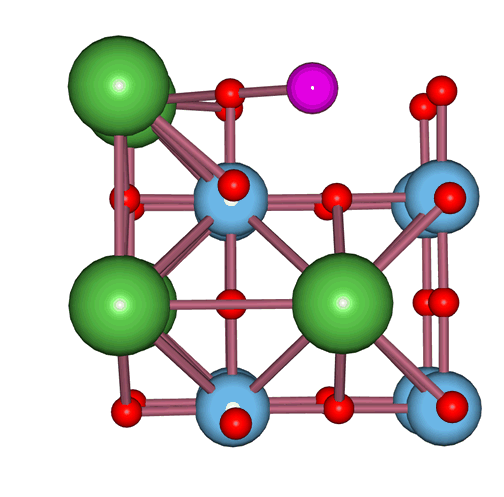

Supplement: Supplementary file 2 — Rotations of the TiO6 octahedra at 62 cm–1. [file 41563_2023_1535_MOESM2_ESM.gif]

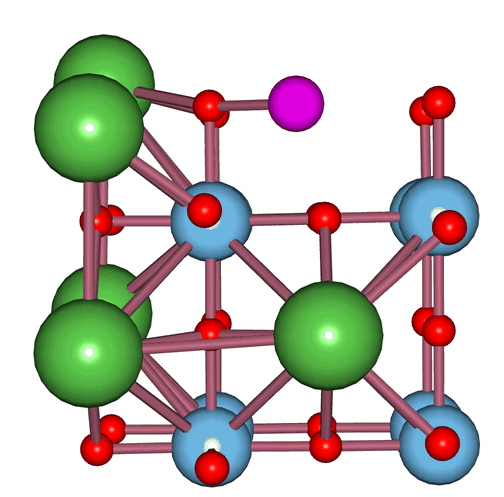

Supplement: Supplementary file 3 — An in-plane optical TO mode at 70 cm–1. [file 41563_2023_1535_MOESM3_ESM.gif]

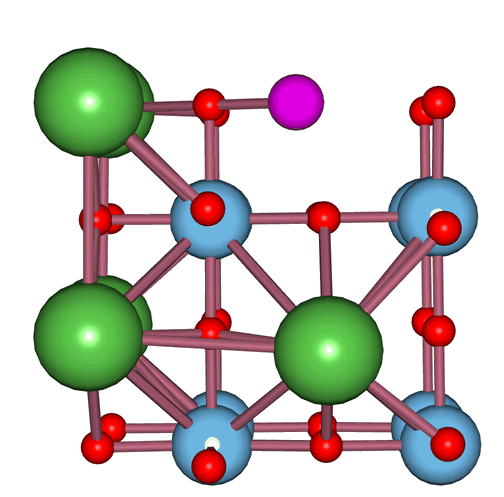

Supplement: Supplementary file 4 — An out-of-plane optical LO mode at 89 cm–1. [file 41563_2023_1535_MOESM4_ESM.gif]

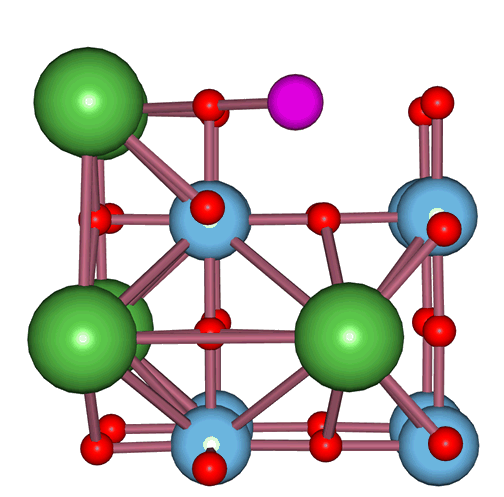

Supplement: Supplementary file 5 — LTO breathing mode at 132 cm–1. [file 41563_2023_1535_MOESM5_ESM.gif]
